# Left ventricular reverse remodeling after aortic valve replacement or repair in bicuspid aortic valve with moderate or greater aortic regurgitation

**DOI:** 10.1016/j.xjon.2024.03.006

**Published:** 2024-03-27

**Authors:** Jonathan D. Kochav, Hiroo Takayama, Andrew Goldstone, David Kalfa, Emile Bacha, Marlon Rosenbaum, Matthew J. Lewis

**Affiliations:** a Division of Cardiology, Department of Medicine, Columbia University Irving Medical Center, New York, NY; b Division of Cardiothoracic and Vascular Surgery, Department of Medicine, Columbia University Irving Medical Center, New York, NY

**Keywords:** bicuspid valve, aortic regurgitation, aortic valve replacement, aortic valve repair, echocardiography

## Abstract

**Objective:**

Bicuspid aortic valve (AV) patients with aortic regurgitation (AR) differ from tricuspid AV patients given younger age, greater left ventricle (LV) compliance, and more prevalent aortic stenosis (AS). Bicuspid AV-specific data to guide timing of AV replacement or repair are lacking.

**Methods:**

Adults with bicuspid AV and moderate or greater AR who underwent aortic valve replacement or repair at our center were studied. The presurgical echocardiogram, and echocardiograms within 3 years postoperatively were evaluated for LV geometry/function, and AV function. Semiquantitative AS/AR assessment was performed in all patients with adequate imaging.

**Results:**

One hundred thirty-five patients (85% men, aged 44.5 ± 15.9 years) were studied (63% pure AR, 37% mixed AS/AR). Following aortic valve replacement or repair, change in LV end-diastolic dimension and change in LV end-diastolic volume were associated with preoperative LV end-diastolic dimension (β = 0.62 Δcm/cm; 95% CI, 0.43-0.73 Δcm/cm; *P* < .001), and LV end-diastolic volume (β = 0.6 ΔmL/mL; 95% CI, 0.4-0.7 ΔmL/mL; *P* < .001), respectively, each independent of AR/AS severity (*P* = not significant). Baseline LV size predicted postoperative normalization (LV end-diastolic dimension: odds ratio, 3.75/cm; 95% CI, 1.61-8.75/cm, LV end-diastolic volume: odds ratio, 1.01/mL; 95% CI, 1.004-1.019/mL, both *P* values < .01) whereas AR/AS severity did not (*P* = not significant). Indexed LV end diastolic volume outperformed LV end-diastolic dimension in predicting postoperative LV normalization (area under the curve = 0.74 vs 0.61) with optimal diagnostic cutoffs of 99 mL/m^2^ and 6.1 cm, respectively. Postoperative indexed LV end diastolic volume dilatation was associated with increased risk of death, transplant/ventricular assist device, ventricular arrhythmia, and reoperation (hazard ratio, 6.1; 95% CI, 1.7-21.5; *P* < .01).

**Conclusions:**

Remodeling extent following surgery in patients with bicuspid AV and AR relates to preoperative LV size independent of valve disease phenotype or severity. Many patients with LV end-diastolic dimension below current surgical thresholds did not normalize LV size. LV volumetric assessment offered superior diagnostic performance for predicting residual LV dilatation, and postoperative indexed LV end diastolic volume dilatation was associated with adverse prognosis.

## Graphical Abstract

**Figure:**
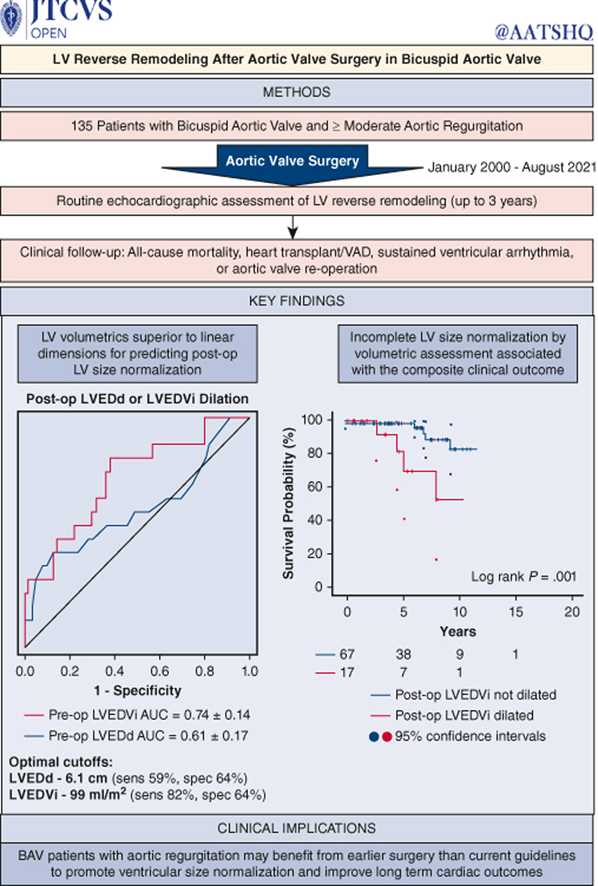

**Figure:**
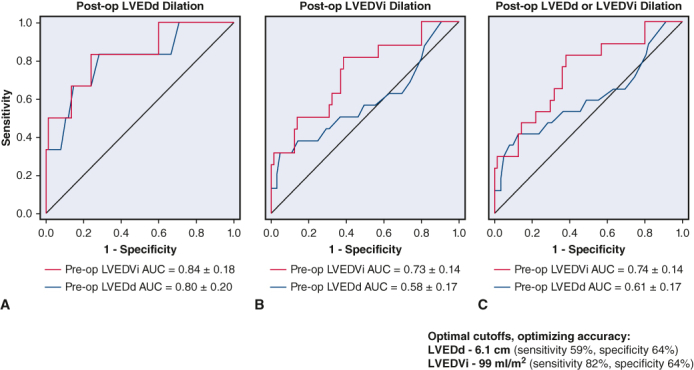
Preop LVEDVi predicts postop LV dilatation with greater accuracy than linear dimensions.

**Central Message**

Bicuspid aortic valve patients with significant regurgitation may benefit from earlier surgery than current guidelines recommend to promote ventricular size normalization and improve cardiac outcomes.

**Perspective**

Bicuspid aortic valve-specific data are required to guide surgical management of regurgitation in these patients. In our retrospective review, we identified linear dimension and volumetric echocardiographic thresholds that predicted LV size normalization after surgery, and were smaller than current guideline recommendations. Failure to normalize LV size was associated with adverse cardiac outcomes.

Bicuspid aortic valve (BAV) is the most common congenital heart defect with a population prevalence of 0.5 to 2%.^1^ BAV degeneration is progressive over time, resulting in valvar stenosis or regurgitation, with BAV patients representing 10 to 40% of all patients with significant regurgitation (AR).^2^^,^^3^ Chronic AR imparts combined left ventricular (LV) volume and pressure loading,^4^ resulting in adverse remodeling with ventricular dilatation, myocyte hypertrophy, fibrosis, and contractile dysfunction.^5^^,^^6^ AV replacement or repair (AVR/r) relieves adverse loading conditions and improves long-term outcomes,^7^^,^^8^ but abnormal LV geometry and function may persist in patients undergoing late repair.^9^^,^^10^

Accordingly, guidelines have been established to trigger early referral for surgery in asymptomatic patients.^11^^,^^12^ US guidelines recommend surgery for LV ejection fraction (LVEF) <55%, LV end-systolic diameter (LVEDs) >5.0 cm, or progressive increase in LV end-diastolic diameter (LVEDd) to >6.5 cm.^11^ However, data supporting these recommendations are derived from cohorts in which BAV patients are generally a minority. BAV patients with AR differ from tricuspid AV patients in several important ways. Firstly, BAV patients are younger at time of incident valve disease with fewer comorbidities.^2^^,^^13^ Correspondingly, increased LV compliance is believed to explain more prominent dilatation in BAV patients,^2^^,^^14^^,^^15^ with nearly half of operations being performed based on increased LVEDd.^2^ Given that a large proportion of patients are followed with, and are referred to surgery for, LV diastolic dilatation, specific BAV-derived data are necessary to understand the LV response to AVR/r with respect to LV diastolic size normalization. Additionally, BAV patients have higher prevalence of concomitant stenosis (AS),^2^^,^^13^ which compounds the hemodynamic load of AR and may also influence reverse remodeling achieved with surgery.

Considering these differences, we sought to assess the degree to which preoperative metrics of ventricular geometry and valve disease phenotype predicted reduction of LV diastolic size following operative intervention, as a marker for long-term postoperative risk in patients with BAV and AR.

## Methods

### Study Population

The population comprised adults (aged 18 years or older) with BAV and moderate or greater AR who underwent AVR/r at a single US medical center between July 2000 and August 2021. Patients with acute AR due to endocarditis were excluded, as were patients with moderate or greater mitral regurgitation or other mechanisms of LV volume loading (eg, ventricular septal defect or patent ductus arteriosus). The echocardiogram before surgery was reviewed, as were echocardiograms at least 3 months but within 3 years postoperatively. Patients with recurrent moderate or greater AR within the study period were excluded from assessments of postoperative reverse remodeling.

Demographic data, including cardiovascular risk factors and medications, were obtained from the medical record and categorized in a uniform manner. This study was conducted with approval of the Institutional Review Board at Columbia University Irving Medical Center (IRB #AAAT2458; approval date: December 4, 2023), which provided approval for analysis of preexisting data.

### Outcomes of Interest

Three primary outcomes of interest were defined: reduction in LV diastolic dimensions following AVR/r; normalization of ventricular size following surgery (LVEDd ≤5.2 cm in women, ≤5.8 cm in men, indexed LV end-diastolic volume [LVEDV] ≤61 mL/m^2^ in women, ≤74 mL/m^2^ in men)^16^; a composite clinical outcome composed of all-cause mortality, heart transplant, or ventricular assist device (VAD); sustained ventricular tachycardia; or AV reoperation.

### Image Acquisition

Two-dimensional transthoracic echocardiograms were performed using commercial equipment. Exams included evaluation of the LV in parasternal long axis and apical^16^ views with and without color Doppler as specified in consensus guidelines.^16^ Evaluation of the right ventricle (RV) outflow tract was performed in parasternal short and long axis. The descending aorta was evaluated from suprasternal and subcostal windows. Continuous-wave Doppler assessment was typically performed to assess transaortic flow velocity profile. Pulsed-wave Doppler assessment was typically performed in the LV and RV outflow tracts, as well as in the descending thoracic aorta.

### Image Analysis

LVEDd and LV end-systolic dimension (LVESd) were measured from the parasternal long axis, and LV end-diastolic and LV end-systolic volumes were quantified via Simpson’s biplane method of disks by a single reader (J.K.) as per the American Society of Echocardiography (ASE) consensus guidelines.^16^ LVEF was assessed qualitatively, as well as quantitatively using the Teicholz and Simpson’s biplane methods. LV size and function was analyzed for the presurgical echocardiogram, and on the postoperative echocardiogram with greatest degree of reverse LV remodeling within the study time frame.

AS severity on the preoperative echocardiogram was ascertained from the clinical report, and then confirmed when able via analysis of transaortic continuous wave Doppler, and LV outflow tract pulsed-wave Doppler waveforms to estimate peak and mean transaortic systolic gradients, aortic valve area, and the dimensionless index.

AR severity on preoperative echocardiogram was ascertained from the clinical report, and then confirmed when adequate imaging was available by a single reader (J.K.) as per the ASE and Society for Cardiovascular Magnetic Resonance joint recommendations.^17^ Vena contracta diameter, and flow convergence size were assessed on color Doppler imaging from parasternal long-axis and apical views. The jet width to LV outflow tract dimension ratio was assessed on color Doppler imaging from the parasternal long-axis view. Prominent diastolic flow reversal in the descending thoracic aorta was assessed by pulsed wave Doppler. Regurgitant volume was estimated by the difference between LV and RV systolic stroke volumes as calculated by outflow tract velocity time integral × area. AR was graded as severe if ≥4 specific criteria, 3 specific criteria with regurgitant volume ≥45 mL or regurgitant fraction ≥40%, or 2 specific criteria were met with regurgitant volume ≥60 mL or regurgitant fraction ≥50%.^17^

### Statistical Analysis

Continuous variables are summarized as means ± SD when normally distributed, and otherwise as medians (interquartile range). Categorical variables are summarized as frequencies and percent. Normally distributed continuous indices were compared via Student t tests (2-group comparisons) or analysis of variance (multiple-group comparisons). Categorical variables were compared using χ^2^ tests or Fisher exact test where appropriate. Pearson’s correlation coefficients were calculated to test associations between preoperative LV size and reverse LV remodeling. Linear regression analysis was used to test associations between AR and AS severity measures with degree of reverse LV remodeling. Logistic regression was used to test predictors of postoperative LV size normalization. Receiver operating characteristics analysis was used to evaluate diagnostic performance of preoperative LV size for prediction of LV size normalization. Kaplan-Meier analysis with log-rank testing and Cox proportional hazards modeling was used to relate postoperative remodeling to clinical outcomes. Statistical calculations were performed using SPSS version 28.0 (IBM-SPSS Inc).

## Results

### Population Characteristics

One hundred thirty-five BAV patients with moderate or greater AR who underwent AVR/r from July 2000 to August 2021 were studied (85% men, mean age, 44.5 ± 15.9 years). Baseline population demographics are shown in Table 1. Eighty-five patients (63%) had pure AR, and 50 patients (37%) had mixed valve disease with at moderate or greater concomitant AS. The majority of patients underwent AVR with a bioprosthetic tissue valve (n = 98 [72%]), followed by similar number of mechanical AVR (n = 15 [11%]) and Ross procedures (n = 16 [12%]), and a smaller number of valve repairs (n = 6 [4%]). Fifty-eight patients (43%) had concomitant aortic repair; for 13 patients (10%), this was the primary indication for surgery. Among this retrospective cohort, 49 patients (36%) met contemporary surgical cutoffs for LVEF ≤55%, 16 (12%) met contemporary thresholds for LVESd ≥5.0 cm, and 32 (24%) for LVEDd ≥6.5 cm. Patients with pure AR were more likely than patients with mixed AS/AR to have met contemporary echocardiographic indications for surgery based on LVEF (48% vs 16%), LVESd (18% vs 2%), and LVEDd (35% vs 4%; all *P* values < .001).

**Table 1:** Population characteristics

Variable	Overall cohort (n = 135)	Dilated postoperative LV size (n = 21)	Normal postoperative LV size (n = 83)	*P* value
Clinical demographics				
Age (y)	44.5 ± 15.9	39.3 ± 14.2	45.3 ± 16.4	.13
Sex				.23
Male	85 (115)	81 (17)	90 (75)	
Female	15 (30)	19 (4)	10 (8)	
BSA (m^2^)	1.96 ± 0.29	1.94 ± 0.26	1.99 ± 0.30	.58
Cardiovascular risk factors				
Hypertension	44 (60)	14 (3)	52 (43)	**<.01**
Hyperlipidemia	28 (38)	14 (3)	32 (26)	.11
Diabetes	6 (8)	0 (0)	10 (8)	.14
Current or previous tobacco use	25 (34)	14 (3)	27 (22)	.23
Family history of premature CAD	10 (14)	5 (1)	10 (8)	.47
No. of risk factors	1.1 ± 1.1	0.5 ± 0.6	1.3 ± 1.2	**<.001**
History of CAD	26 (35)	14 (3)	28 (23)	.20
Prior myocardial infarction	4 (5)	0 (0)	1 (1)	1.00
Prior revascularization	5 (7)	5 (1)	5 (4)	1.00
Heart failure medications at follow-up				
Beta-blocker	41 (55)	55 (11)	51 (42)	.76
ACE inhibitor/ARB	30 (40)	35 (7)	39 (32)	.74
Aldosterone antagonist	3 (4)	15 (3)	1 (1)	**.02**
Surgical characteristics				
Type of valve surgery				.60
Mechanical AVR	11 (15)	14 (3)	10 (8)	
Tissue AVR	72 (98)	62 (13)	74 (61)	
Ross	12 (16)	14 (3)	14 (11)	
Valve repair	4 (6)	10 (2)	4 (3)	
Concomitant thoracic aneurysm repair	43 (58)	48 (10)	45 (37)	.80
Concomitant coronary artery bypass	6 (8)	5 (1)	6 (5)	1.00
Surgical indication				.62
Pure aortic regurgitation	63 (85)	62 (13)	57 (47)	
Mixed aortic regurgitation and stenosis	37 (50)	23 (5)	34 (28)	
Aneurysm repair as primary indication	10 (13)	14 (3)	10 (8)	
Prior aortic valve repair	8 (11)	9 (2)	8 (7)	1.00

Values are presented as mean ± SD or % (n). Boldface type reflects *P* value <.05. *LV*, Left ventricle; *BSA*, body surface area; *CAD*, coronary artery disease; *ACE*, angiotensin-converting enzyme; *ARB*, angiotensin-receptor blocker; *AVR*, aortic valve replacement.

Based on clinical echocardiographic reports, AR severity was categorized as moderate (n = 48 [36%]), moderate-severe (n = 48 [36%]), or severe (n = 39 [29%]). AS severity was categorized as none (n = 84 [62%]), mild (n = 23 [17%]), moderate (n = 10 [7%]), or severe (n = 18 [13%]). Sufficient imaging was available for a complete Doppler-based assessment in 98 patients (73%). Relation of the clinical report to semi-quantitative and quantitative parameters is shown in Tables E1 and E2; each color and quantitative Doppler-based measure of AR was associated with the initial assessment.

### Baseline LV Geometry in Relation to AV Disease

Preoperative parameters of LV geometry are depicted in Tables 2 and 3, stratified by clinical report and ASE algorithm-derived AR severity (Table 2), and by valve disease phenotype (pure AR vs mixed disease) (Table 3). Patients with greater AR had a greater degree of LV remodeling, as evidenced by increased LV size and lower LVEF. Similarly, patients with pure AR had greater LV size and lower LVEF compared with mixed valve disease patients.

**Table 2:** Left ventricular geometry stratified by aortic regurgitation (AR) severity

Variable	Initial assessment of AR severity					ASE algorithm derived AR severity			
	Overall (N = 135)	Moderate (n = 48)	Moderate-severe (n = 48)	Severe (n = 39)	*P* value	Overall (n = 98)	Moderate (n = 35)	Severe (n = 63)	*P* value
Dimensions									
LV end-diastolic dimension (cm)	5.92 ± 0.84	5.49 ± 0.70	6.07 ± 0.80	6.28 ± 0.87	**<.001**	5.73 ± 0.76	5.20 ± 0.54	6.04 ± 0.68	**<.001**
LV end-systolic dimension, cm	3.92 ± 0.96	3.45 ± 0.83	4.10 ± 0.95	4.30 ± 0.90	**<.001**	3.85 ± 1.00	3.34 ± 0.58	4.13 ± 1.06	**<.001**
LVEF, Teicholz (%)	61.0 ± 13.6	64.4 ± 13.1	59.1 ± 15.1	59.2 ± 11.8	.11	59.7 ± 14.6	63.5 ± 11.8	57.6 ± 15.6	.05
LV mass (g)	282.2 ± 93.2	263.2 ± 84.5	287.9 ± 90.4	298.4 ± 104.7	.20	272.0 ± 95.6	242.4 ± 74.3	288.0 ± 102.3	**.02**
LV mass, index (g/m^2^)	143.7 ± 44.0	133.3 ± 36.0	147.2 ± 47.5	151.8 ± 46.8	.13	138.6 ± 42.5	124.7 ± 34.3	146.0 ± 44.8	**.02**
Relative wall thickness	0.38 ± 0.11	0.42 ± 0.12	0.36 ± 0.08	0.35 ± 0.12	**.01**	0.39 ± 0.12	0.44 ± 0.13	0.36 ± 0.10	**<.001**
Qualitative LVEF (%)	55.9 ± 9.8	58.4 ± 8.1	54.4 ± 11.4	54.5 ± 9.2	.08	55.8 ± 11.1	59.1 ± 9.9	54.0 ± 11.3	**0.03**
Simpsons biplane parameters									
LV end-diastolic volume (mL)	189.0 ± 78.8	150.8 ± 67.7	207.1 ± 75.5	221.2 ± 77.6	**<.001**	188.4 ± 80.5	130.1 ± 46.9	222.2 ± 76.7	**<.001**
LV end-diastolic volume, index (mL/m^2^)	96.1 ± 37.6	75.3 ± 28.6	106.5 ± 37.7	113.0 ± 35.9	**<.001**	96.3 ± 38.5	67.2 ± 21.4	113.3 ± 36.0	**<.001**
LV end-systolic volume (mL)	86.4 ± 53.0	63.7 ± 38.9	102.6 ± 56.4	98.8 ± 56.3	**<.01**	85.8 ± 54.2	53.5 ± 29.4	104.7 ± 56.6	**<.001**
LV end-systolic volume, index (mL/m^2^)	43.8 ± 25.8	31.6 ± 16.9	52.7 ± 29.3	50.2 ± 25.5	**<.001**	43.7 ± 26.4	27.4 ± 13.8	53.2 ± 27.4	**<.001**
LVEF (%)	56.6 ± 11.3	59.6 ± 11.1	52.9 ± 11.2	57.0 ± 10.6	**.04**	56.9 ± 11.0	61.0 ± 11.1	54.5 ± 11.0	**<.01**
LV dilatation	81 (109)	63 (30)	92 (44)	90 (35)	**<.001**	80 (78)	49 (17)	98 (61)	**<.001**
LV dilated by dimensions	60 (81)	40 (19)	71 (34)	72 (28)	**<.01**	52 (51)	20 (7)	70 (44)	**<.001**
LV dilated by volumes	56 (75)	49 (19)	91 (31)	93 (25)	**<.001**	71 (70)	40 (14)	93 (56)	**<.001**

Values are presented as mean ± SD or n (%). Boldface type reflects *P* value <.05. *ASE*, American Society of Echocardiography; *LV*, left ventricle; *LVEF*, left ventricular ejection fraction.

**Table 3:** Left ventricular geometry stratified by valve disease phenotype

Variable	Valve disease phenotype			
	Overall (N = 135)	Pure AR (n = 85)	Mixed AS/AR (n = 50)	*P* value
Dimension				
LV end-diastolic dimension (cm)	5.92 ± 0.84	6.25 ± 0.73	5.36 ± 0.73	**<.001**
LV end-systolic dimension (cm)	3.92 ± 0.96	4.20 ± 0.91	3.46 ± 0.86	**<.001**
LV ejection fraction, Teicholz (%)	61.0 ± 13.6	59.1 ± 13.4	64.2 ± 13.5	**.04**
LV mass (g)	282.2 ± 93.2	292.5 ± 96.7	265.6 ± 85.7	.11
LV mass index (g/m^2^)	143.7 ± 44.0	146.9 ± 46.5	138.7 ± 39.7	.31
Relative wall thickness	0.38 ± 0.11	0.34 ± 0.08	0.44 ± 0.13	**<.001**
Qualitative LV ejection fraction (%)	55.9 ± 9.8	53.8 ± 8.8	59.4 ± 10.5	**.001**
Simpsons biplane parameters				
LV end-diastolic volume (mL)	189.0 ± 78.8	217.9 ± 76.2	150.6 ± 65.3	**<.001**
LV end-diastolic volume index (mL/m^2^)	96.1 ± 37.6	109.6 ± 36.9	78.2 ± 30.6	**<.001**
LV end-systolic volume (mL)	86.4 ± 53.0	104.2 ± 50.6	62.9 ± 47.0	**<.001**
LV end-systolic volume index (mL/m^2^)	43.8 ± 25.8	52.1 ± 23.8	32.7 ± 24.3	**<.001**
LV ejection fraction (%)	56.6 ± 11.3	53.2 ± 9.8	61.2 ± 11.7	**<.001**
LV dilatation	81 (109)	91 (77)	64 (32)	**<.001**
LV dilated by dimensions	60 (81)	75 (64)	34 (17)	**<.001**
LV dilated by volume	56 (75)	93 (53)	51 (22)	**<.001**

Values are presented as mean ± SD or % (n). Boldface type reflects *P* value <.05. *AS*, Aortic stenosis; *LV*, left ventricle.

### Reverse LV Remodeling Following AVR/r

**Figure 1:**
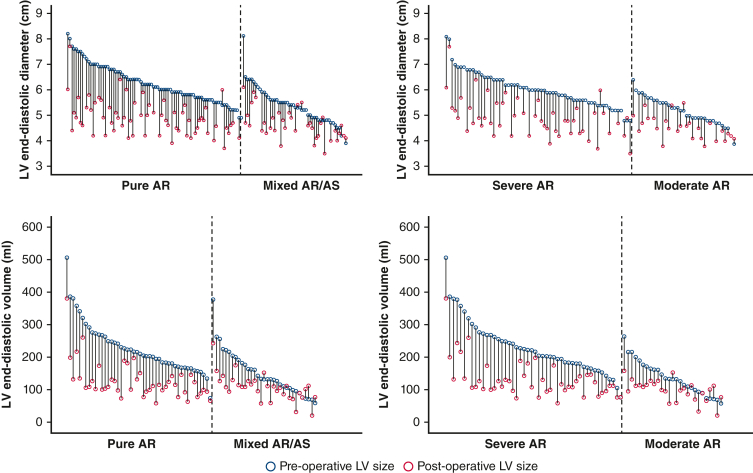
Reverse remodeling of left ventricle (*LV*) size in relation preoperative LV size, and in relation to valve disease phenotype (*left*) and AR severity (*right*). Each line reflects size change in individual patients, presented in descending order of baseline size. LV size is characterized by LV end-diastolic dimensions (*top*) and LV end-diastolic volume (*bottom*). *AR*, Aortic regurgitation; *AS*, aortic stenosis.

**Figure 2:**
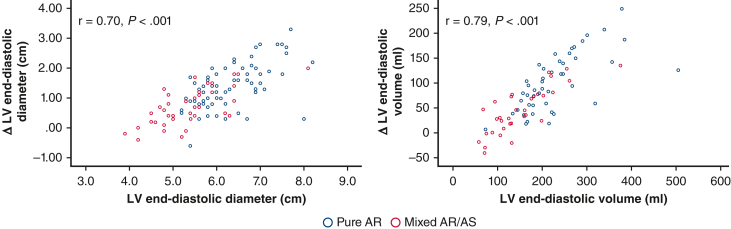
Scatterplots relating post-operative change in left ventricle (*LV*) end-diastolic dimensions to preoperative LV end-diastolic dimensions (*left*), and postoperative change LV end-diastolic volume to preoperative LV end-diastolic volume. Plots are stratified by valve disease phenotype (pure aortic regurgitation [*AR*] [*blue*] vs mixed AR/aortic stenosis [*AS*] [*red*]).

One hundred five patients (78%) had both pre- and post-AVR/r echocardiograms available for review. One patient who underwent repair developed moderate or greater AR within the study period and was excluded from further analyses. Mean time to echocardiogram with greatest reverse remodeling was 1.9 ± 1.1 years. The plots in Figure 1 depict patient level data for pre- and post-AVR/r LV size stratified by valve disease phenotype and by ASE-algorithm derived AR severity. As shown, ΔLVEDd (1.41 ± 0.76 cm vs 0.71 ± 0.63 cm) and ΔLVEDV (101.0 ± 56.8 mL vs 44.2 ± 45.8 mL; both *P* values < .001) were each greater for patients with pure AR versus mixed AS/AR. ΔLVEDd (1.19 ± 0.67 cm vs 0.62 ± 0.52 cm; *P* < .001) and ΔLVEDV (98.1 ± 59.4 mL vs 38.4 ± 41.2 mL; *P* < .001) were each significantly greater for patients with severe versus moderate AR. Related scatterplots in Figure 2 depict ΔLVEDd and ΔLVEDV post-AVR/r against preoperative LV size, stratified by valve disease phenotype. Both ΔLVEDd (*r* = 0.70; *P* < .001), and ΔLVEDV (*r* = 0.79; *P* < .001) were strongly correlated with preoperative LV size. In multivariable analyses, the relationship between baseline LV size to reverse remodeling was independent of valve disease phenotype, which was not predictive (*P* = not significant) (Table E3).

Linear regression analysis was performed to assess predictors of LV reverse remodeling following AVR/r (Table 4). In the overall cohort (Models 1 and 2), AR severity predicted postoperative ΔLVEDd (β = 0.30 cm per half-grade; 95% CI, 0.11 to 0.50; *P* < .01), independently of AS severity (β = −0.14 cm per grade; 95% CI, −0.30 to −0.00; *P* = .04). However, postoperative ΔLVEDd was most strongly associated with preoperative LVEDd, which predicted ΔLVEDd (β = 0.58 cm per preoperative LVEDd; 95% CI, 0.43 to 0.73; *P* < .001) independent of both AR and AS severity (both *P* values = not significant).

**Table 4:** Predictors of left ventricular (LV) reverse remodeling following aortic valve replacement or repair (AVR/r)

Variable∗	Univariable β	95% CI	*P* value	Multivariable β	95% CI	*P* value
Overall cohort						
Model 1 – Δ LVEDd						
AR severity, per half-grade increase (cm)	0.39	0.21 to 0.57	**<.001**	0.30 cm	0.11 to 0.50	**<.01**
AS severity, per grade increase (cm)	−0.25	−0.39 to −0.11	**<.001**	−0.15 cm	−0.30 to −0.00	**.04**
Model 2 – Δ LVEDd						
AR severity, per half-grade increase (cm)	0.39	0.21 to 0.57	**<.001**	0.10	−0.06 to 0.26	.23
AS severity, per grade increase (cm)	−0.25	−0.39 to −0.11	**<.001**	0.01	−0.12 to 0.13	.88
LVEDd (per cm)	0.62	0.49 to 0.74	**<.001**	0.58	0.43 to 0.73	**<.001**
Complete imaging available						
Model 3 – Δ LVEDV						
Severe AR, vs moderate (mL)	59.7	34.0 to 85.4	**<.001**	46.3	17.5 to 75.2	**<.01**
AS severity, per cm^2^ aortic valve area decrement (mL)	−20.7	−31.4 to −9.9	**<.001**	−12.1	−23.9 to −0.4	**.04**
Model 4 – Δ LVEDV						
Severe AR, vs moderate (mL)	59.7	34.0 to 85.4	**<.001**	11.9	−10.2 to 34.0	.29
AS severity, per cm^2^ aortic valve area decrement (mL)	−20.7	−31.4 to −9.9	**<.001**	4.3	−4.9 to 13.6	.35
LVEDV (per mL)	0.6	0.46 to 0.66	**<.001**	0.6	0.4 to 0.7	**<.001**
Model 5 – Δ LVEDV						
AR severity, No. specific criteria, per criterion (mL)	32.3	6.1 to 58.6	**<.001**	5.3	−0.3 to 10.8	.06
AS severity, per cm^2^ aortic valve area decrement (mL)	−20.7	−31.4 to −9.9	**<.001**	4.4	−4.5 to 13.3	.33
LVEDV (per mL)	0.6	0.5 to 0.7	**<.001**	0.6	0.4 to 0.7	**<.001**
Model 6 – Δ LVEDV						
AR severity, regurgitant volume, per mL	1.2	0.7 to 1.67	**<.001**	0.4	0.0 to 0.9	**<.05**
AS severity, per cm^2^ aortic valve area decrement	−20.7	−31.4 to −9.9	**<.001**	7.9	−2.0 to 17.8	.11
LVEDV, per mL	0.6	0.5 to 0.7	**<.001**	0.6	−0.5 to 0.7	**<.05**

Boldface type reflects *P* value <.05. *LVEDd*, Left ventricular end-diastolic dimension; *AR*, aortic regurgitation; *AS*, aortic stenosis; *LVEDV*, left ventricular end diastolic volume.

∗ All variables included in the multivariable analyses are displayed in each respective model.

Similar findings were seen among the cohort of patients with complete imaging available (Table 4, Models 3-6). Severe AR predicted ΔLVEDV (β = 46.3 mL; 95% CI, 17.5 to 75.2; *P* < .01), independent of AS severity (β = −12.8 mL/cm^2^ AV area decrement; 95% CI, −23.9 to −0.4; *P* = .04). Whereas preoperative LVEDV predicted ΔLVEDV (β = 0.6 mL per preoperative LVEDV; 95% CI, 0.4 to 0.7; *P* < .001) independent of both AS and AR severity (both *P* values = not significant), there was a signal for independent predictive power of AR severity when analyzed on a continuum (number of severe AR criteria or regurgitant volume) as shown in models 5 and 6.

Valve type was also tested as a determinant of reverse remodeling. Against a reference of tissue AVR, Ross patients had greater ΔLVEDd (β = 0.49 cm; 95% CI, 0.05 to 0.94; *P* = .03) and AVr patients had a trend toward lesser ΔLVEDd (β = −0.66 cm; 95% CI, −1.36 to 0.04; *P* = .07), with no difference seen following mechanical AVR (β = −0.20 cm; 95% CI, −0.69 to 0.29; *P* = .43). Notably, repair patients comprised the smallest cohort (n = 6) and included the only patient with moderate or greater postoperative AR (excluded from analysis), and the only patient with major intraoperative myocardial infarction. Also, whereas different surgical approaches are expected to yield differences in transvalvular systolic velocities, peak postoperative AV gradient was not associated with ΔLVEDd (*P* = .29).

### Predictors of Failure to Normalize LV Size

Twenty-one patients (16% of patients with postoperative imaging) failed to achieve LV size normalization, with 18 (13%) dilated based on volumetric criteria and 11 (8%) by dimensions. As shown in Table 1, patients with postoperative residual LV dilatation were similar in age (39.3 ± 14.2 vs 45.3 ± 16.4 years; *P* = .13), with significantly fewer cardiovascular risk factors (0.5 ± 0.6 vs 1.3 ± 1.2; *P* < .001). Among the overall cohort, increased preoperative LVEDd was associated with failure of LV size normalization after surgery (odds ratio, 3.75 per cm; 95% CI, 1.61-8.75; *P* < .01), whereas valve disease phenotype (pure AR vs mixed AS/AR), AR severity, and AS severity were not (*P* = not significant for each in univariable analysis). Similarly, in the cohort of patients with complete imaging available, LVEDV was associated with failure of LV size normalization (odds ratio, 1.01 per mL; 95% CI, 1.004-1.019; *P* < .01), whereas severe AR, number of specific criteria for severe AR regurgitant volume, and AS severity were not (*P* = not significant for each).

**Figure 3:**
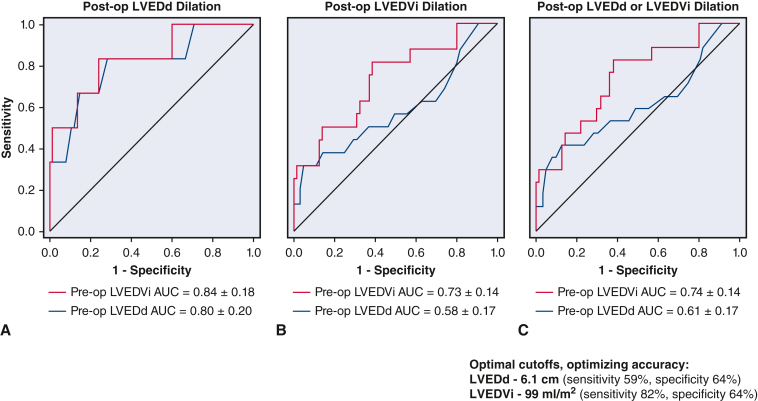
Diagnostic performance of preoperative indexed left ventricular end-diastolic volume (*LVEDVi*) (*red*) and left ventricular end-diastolic dimension (*LVEDd*) (*blue*), for predicting postoperative residual LV dilatation as based on postoperative LVEDd (A), LVEDVi (B), or both (C).

Receiver operating characteristics analysis was performed to evaluate diagnostic performance of LVEDd and indexed LVEDV (LVEDVi) for predicting postoperative LV size normalization (Figure 3). Both LVEDVi and LVEDd performed similarly in predicting persistently elevated LVEDd (area under the curve, 0.84 vs 0.80) (Figure 3, *A*). However, LVEDVi outperformed LVEDd in predicting persistently elevated LVEDVi (area under the curve, 0.73 vs 0.58) (Figure 3, *B*), or persistent LV dilatation determined by either dimensions or volumes (area under the curve, 0.74 vs 0.61) (Figure 3, *C*). For LVEDd, optimal cutoff for diagnostic accuracy was 6.1 cm, yielding sensitivity of 59% and specificity of 64% for postoperative LV dilatation. For LVEDVi, the optimal cutoff was 99 mL/m^2^, yielding sensitivity of 82% and specificity of 64%. No patient with LVEDd ≥8.0 cm or LVEDVi ≥165 mL/m^2^ had postoperative normalization of LV volumes.

### Clinical Prognosis

**Figure 4:**
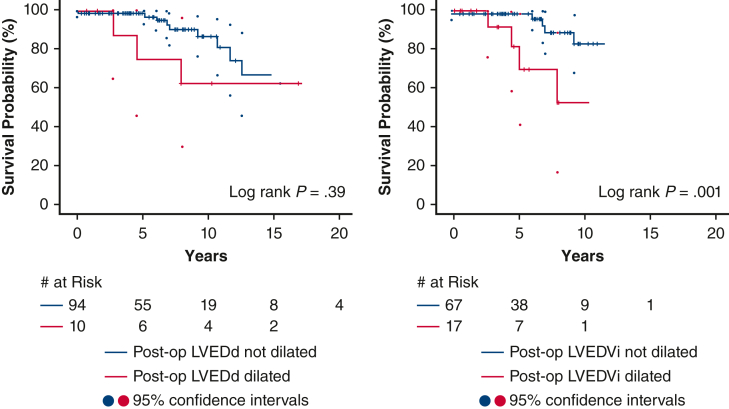
Kaplan-Meier survival curves depicting a composite outcome of all-cause mortality, heart transplant, sustained ventricular arrhythmia, and aortic valve reoperation. Patients were stratified based on postoperative residual left ventricle (*LV*) dilatation based on linear dimensions (*LVEDd*) (*left*), or volumetric analysis (*LVEDVi*) (*right*). Although outcome-free survival was impaired patients with postoperatively elevated LVEDVi (*P* = .001), increased LVEDd was not associated with the composite outcome (*P* = not significant).

All-cause mortality, heart transplant or VAD, sustained ventricular tachycardia, and AV reoperation were assessed to determine whether failure to normalize LV size predicted adverse events. Over a median follow-up of 6.3 years (interquartile range, 3.7-9.5 years), 13 events occurred (13%). Events included death or transplant/VAD in 4 patients (4%; 3 deaths, 1 VAD), ventricular arrhythmia in 3 patients (3%) and AV reoperation in 8 patients (8%). Notably, all AV reoperations were following tissue AVR, for symptomatic prosthetic valve dysfunction in 7 and prosthetic valve endocarditis in 1. Kaplan-Meier analysis was performed to assess postoperative LV dilatation in relation to clinical outcomes (Figure 4). Whereas postoperative increased LVEDd was not associated with the composite clinical outcome (hazard ratio [HR], 1.8; 95% CI, 0.5-6.5; *P* = .40), postoperative LVEDVi dilation was (HR, 6.1; 95% CI, 1.7-21.5; *P* < .01). LVEDVi dilatation remained associated with adverse events when aortic valve reoperation (HR, 5.0; 95% CI, 1.0-24.9; *P* = .05) or ventricular arrhythmia (HR, 4.6; 95% CI, 1.0-21.0; *P* = .05) were excluded from the composite outcome.

## Discussion

**Figure 5:**
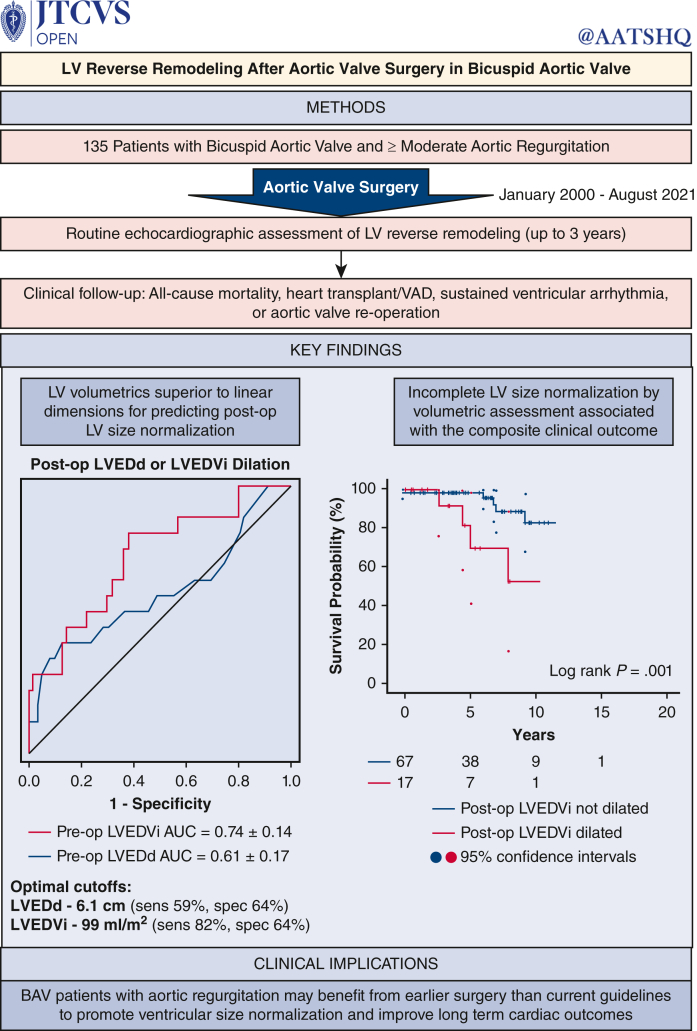
Graphical abstract. *LV*, Left ventricle; *VAD*, ventricular assist device; *LVEDd*, left ventricular end-diastolic dimension; *LVEDVi*, left ventricular end diastolic volume; *AUC*, area under the curve; *BAV*, bicuspid aortic valve.

This study yields novel insights regarding LV remodeling in relation to preoperative LV geometry and valve disease phenotype among BAV patients with moderate or greater AR undergoing AVR/r (Figure 5). First, extent of reverse remodeling following surgery related to preoperative LV size independent of valve disease phenotype or echocardiogram-derived valve disease severity. Second, LV volumetric assessment offered superior diagnostic performance for predicting residual LV dilatation compared with LV dimensions. Finally, incomplete LV reverse remodeling by volumetrics was associated with increased risk of a composite outcome of death, transplant/VAD, sustained ventricular arrhythmia, and aortic valve reoperation.

Many prior studies have investigated the influence of AR on LV remodeling, and the degree to which reverse remodeling occurs after surgery, to better guide timing of AVR/r.^11^^,^^12^ However specific data are lacking to guide expectation of reverse remodeling among BAV patients. There are reasons to believe that the BAV population is unique and that extrapolation of data derived from all-cause AR may not be appropriate. Due to younger age at incident valve disease and greater relative LV compliance, BAV patients with pure AR frequently develop a greater degree of LV dilatation without triggering symptomatic or systolic thresholds for surgery.^2^ Additionally, younger age at surgery, and prevalent use of strategies with suboptimal durability (eg, tissue AVR and valve repair)^18^^,^^19^ result in high likelihood of recurrent valvulopathy-related myocardial injury over the lifetime. Thus, selection of valve substitutes with improved durability, and demonstrating relative mortality benefit in young patients (eg, mechanical valve^20^ or pulmonary autograft^21^), as well as establishing a more aggressive threshold for intervention to minimize postoperative residual LV dilation, may be appropriate to decrease long-term morbidity. Recent data in tetralogy of Fallot patients with pulmonary regurgitation demonstrate improved long-term outcomes with a proactive approach for valve replacement,^22^ supporting the notion that delaying intervention on valvular regurgitation beyond the point of adaptive and reversible myocardial remodeling can translate to inferior clinical outcomes.

Prior studies in AR populations have demonstrated that increased LV diastolic size is associated with adverse events,^15^ and that delaying surgery to extremes of LV dilatation can result in failure to normalize LV size and unfavorable surgical outcomes,^23^^,^^24^ prompting guideline recommendations for surgery when LVEDd progressively increases to >6.5 cm. In our study, an LVEDd of 6.1 cm provided highest diagnostic accuracy for predicting residual dilatation, indicating that surgery at lower thresholds may optimize long-term myocardial protection, although further study is needed to link LV size normalization with improved clinical outcomes. Such findings are consistent with emerging data supporting earlier surgery in BAV patients.^2^^,^^25^ For example, Hecht and colleagues^25^ recently demonstrated all-cause mortality to be increased once LVEF drops below 60%, values above contemporary guidelines for surgical intervention.^11^^,^^12^

Beyond excess LV remodeling, BAV patients with AR also differ from tricuspid AV patients with respect to increased prevalence of concomitant AS. Despite accounting for a minority of AR patients,^2^^,^^3^^,^^13^ BAV represent up to half of patients with mixed AV disease,^26^ in whom co-existing AS compounds adverse loading conditions imparted by AR.^27^ Egbe and colleagues^28^ showed that patients with moderate mixed AV disease had worse clinical outcomes, comparable to severe AS patients, and Philip and colleagues^29^ demonstrated that AR patients with more severe AS have reduced survival following AVR. Patients with mixed AS/AR are also more likely to have surgery based on symptoms than on meeting echocardiographic criteria.^28^^,^^30^ However, how concomitant AS influences reverse remodeling after AVR has not been well defined. In our study, valve disease phenotype and degree of AS were not associated with reverse remodeling after AVR controlling for baseline LV size. These findings support current valve guidelines^11^^,^^12^ that indicate that influence of AS and AR should be considered independently. Other factors beyond chamber size, such as progressive concentric remodeling and diastolic dysfunction may play an important role and should drive consideration of early valve surgery in patients with mixed valve disease.^31^

It is important to note that although current guidelines recommend surgical referral based on linear dimensions, volumetric analysis is the standard of care for LV chamber quantification,^16^ and likely better reflects spherical remodeling that occurs in AR.^32^ In our study, LV volumes were more sensitive for identifying postoperative dilatation (13% vs 8%), and also had greater diagnostic accuracy for predicting postoperative LV size normalization (area under the curve, 0.71 vs 0.57) and clinical outcomes. Akintoye and colleagues^15^ recently demonstrated that echocardiographic volumetric assessment had greater prognostic value than linear dimensions in >500 AR patents, and identified optimal age and sex-related cutoffs for predicting adverse outcomes, with cutoffs for young men (age younger than 60 years) closely approximating those identified in our study for predicting incomplete reverse remodeling (94 vs 99 mL/m^2^). Such findings support the concept that volumetrics should supplant dimensions for surveillance of LV remodeling, and that advanced techniques such as cardiac magnetic resonance may have increased utility in surveillance. Recent studies have related cardiac magnetic resonance-derived volumetrics with symptoms and outcomes^33^^,^^34^; Malahfji and colleagues^34^ studied 458 patients with asymptomatic moderate or greater AR and preserved ejection fraction, and defined volumetric thresholds (LVEDVi >109 mL/m^2^ and indexed LV end-systolic volume >43 mL/m^2^) more sensitive than linear dimensions for predicting clinical events.

Several limitations should be noted. Sample size was limited due to the single-center nature of this study, and due to the wide era of patients studied, complete Doppler assessment was not possible in all patients. However, semiquantitative and quantitative parameters were strongly associated with the clinical assessments of AV disease, and analyses of semiquantitative AR and AS severity in relation to reverse LV remodeling corresponded closely to findings based on qualitative assessments in the overall cohort. The weak relationship between AR severity and reverse LV remodeling when accounting for baseline LV size may be partially related to limitations in ascertainment of AR severity and LV volumes by echocardiography, and findings may have been different if more precise techniques such as phase contrast and cine-cardiac magnetic resonance were utilized to quantify AR and LV remodeling, respectively.^35^ On the other hand, echocardiography remains the standard of care in the assessment of AR patients, and our findings reflect the data obtained in current practice. Although the echocardiogram with greatest degree of LV remodeling within the first 3 years following AVR was used, clinical follow-up was not standardized and differed among patients, and thus the greatest degree of LV remodeling may have been uncaptured in some patients. Patients in this cohort were overwhelmingly men, precluding assessments of sex-specific differences in LV remodeling. Finally, the number of clinical outcomes was limited in this cohort, precluding multivariable analyses.

## Conclusions

This study demonstrates extent of reverse remodeling following surgery for BAV with AR to relate primarily to preoperative LV size independent of valve disease phenotype or echocardiogram-derived valve disease severity. Many patients with LV diastolic dimensions less than current guideline thresholds for surgery did not have complete normalization of LV size, and LV volumetric assessment offered superior diagnostic performance for predicting postoperative residual LV dilatation, as well as adverse outcomes following incomplete reverse remodeling. Further longitudinal imaging studies using advanced techniques are necessary to define optimal geometric thresholds to predict normalization of LV size following AVR/r in BAV, and to confirm whether or not LV size normalization confers improved freedom from adverse clinical outcomes.

## Conflict of Interest Statement

The authors reported no conflicts of interest.

The *Journal* policy requires editors and reviewers to disclose conflicts of interest and to decline handling manuscripts for which they may have conflict of interest. The editors and reviewers of this article have no conflicts of interest.

## Figures and Tables

**Table E1:** Clinical report-derived aortic regurgitation (AR) severity in relation to semiquantitative parameters

Variable	Overall (N = 135)	Moderate (n = 48)	Moderate-severe (n = 48)	Severe (n = 39)	*P* value
Specific criteria for severe AR					
Vena contracta (cm)	0.67 ± 0.16	0.56 ± 0.12	0.67 ± 0.11	0.82 ± 0.14	**<.001**
Jet width/LVOT dimension ratio	0.67 ± 0.16	0.59 ± 0.14	0.68 ± 0.14	0.77 ± 0.18	**<.001**
Pressure half time (msec)	407 ± 135	459 ± 113	376 ± 123	372 ± 156	**<.01**
Large flow convergence	53 (52)	29 (11)	56 (19)	85 (22)	**<.001**
Prominent diastolic flow reversal in descending aorta	65 (75)	36 (15)	78 (31)	88 (29)	**<.001**
Dilated LV with normal function	60 (81)	50 (24)	69 (33)	62 (24)	.168
No. of specific criteria	1.8 ± 1.4	3.3 ± 1.4	4.2 ± 1.3	4.2 ± 1.3	**<.001**
Quantitative criteria					
Regurgitant volume (mL)	64 ± 28	46 ± 20	68 ± 29	84 ± 22	**<.001**
Regurgitant fraction (%)	46 ± 15	36 ± 14	48 ± 11	57 ± 9	**<.001**

Values are presented as mean ± SD or n (%). Boldface type reflects *P* value <.05. *LVOT*, Left ventricular outflow tract; *LV*, left ventricle.

**Table E2:** Clinical report-derived aortic stenosis severity in relation to semiquantitative parameters

Variable	None (n = 84)	Mild (n = 23)	Moderate (n = 10)	Severe (n = 18)	*P* value
Quantitative criteria					
Peak gradient (mm Hg)	23.1 ± 11.3	60.6 ± 18.9	75.8 ± 25.4	97.2 ± 32.0	<.001
Mean gradient (mm Hg)	13.0 ± 6.9	34.0 ± 8.8	43.5 ± 14.6	54.6 ± 16.9	<.001
Aortic valve area (cm^2^)	2.90 ± 1.04	1.43 ± 0.45	1.18 ± 0.27	0.98 ± 0.40	<.001
Dimensionless index	0.51 ± 0.15	0.31 ± 0.07	0.26 ± 0.04	0.22 ± 0.07	<.001

Values are presented as mean ± SD.

**Table E3:** Left ventricle (LV) reverse remodeling in relation to baseline LV size and valve disease phenotype

Overall cohort						
Variable	Univariable β	95% CI	*P* value	Multivariable β	95% CI	*P* value
Model 1 – Δ LVEDd						
LVEDd (per cm)	0.62	0.49 to 0.74	**<.001**	0.57	0.43 to 0.72	**<.001**
Mixed valve disease (cm)	−0.68	−0.97 to −0.39	**<.001**	−0.15	−0.42 to 0.12	.26
Model 2 – Δ LVEDd						
LVEDV (per mL)	0.6	0.5 to 0.7	**<.001**	0.5	0.4 to 0.6	**<.001**
Mixed valve disease (mL)	−56.8	−80.7 to −32.9	**<.001**	17.0	−35.7 to 1.6	.07

Boldface type reflects *P* value <.05. *LVEDd*, Left ventricular end-diastolic dimension; *LVEDV*, left ventricular end-diastolic volume.
